# SNPs, short tandem repeats, and structural variants are responsible for differential gene expression across C57BL/6 and C57BL/10 substrains

**DOI:** 10.1016/j.xgen.2022.100102

**Published:** 2022-03-09

**Authors:** Milad Mortazavi, Yangsu Ren, Shubham Saini, Danny Antaki, Celine L. St. Pierre, April Williams, Abhishek Sohni, Miles F. Wilkinson, Melissa Gymrek, Jonathan Sebat, Abraham A. Palmer

**Affiliations:** 1 Department of Psychiatry, University of California San Diego, La Jolla, CA, USA; 2 Department of Computer Science and Engineering, University of California San Diego, La Jolla, CA, USA; 3 Department of Cellular and Molecular Medicine and Pediatrics, University of California San Diego, La Jolla, CA, USA; 4 Department of Genetics, Washington University School of Medicine, St. Louis, MO, USA; 5 Salk Institute for Biological Studies, La Jolla, CA, USA; 6 Department of Obstetrics, Gynecology and Reproductive Sciences, University of California San Diego, La Jolla, CA, USA; 7 Institute for Genomic Medicine, University of California San Diego, La Jolla, CA, USA; 8 Department of Medicine, University of California San Diego, La Jolla, CA, USA; 9 Lead contact

## Abstract

Mouse substrains are an invaluable model for understanding disease. We compared C57BL/6J, which is the most commonly used inbred mouse strain, with eight C57BL/6 and five C57BL/10 closely related inbred substrains. Whole-genome sequencing and RNA-sequencing analysis yielded 352,631 SNPs, 109,096 indels, 150,344 short tandem repeats (STRs), 3,425 structural variants (SVs), and 2,826 differentially expressed genes (DE genes) among these 14 strains; 312,981 SNPs (89%) distinguished the B6 and B10 lineages. These SNPs were clustered into 28 short segments that are likely due to introgressed haplotypes rather than new mutations. Outside of these introgressed regions, we identified 53 SVs, protein-truncating SNPs, and frameshifting indels that were associated with DE genes. Our results can be used for both forward and reverse genetic approaches and illustrate how introgression and mutational processes give rise to differences among these widely used inbred substrains.

## In brief

Mortazavi et al. identify polymorphisms (SNPs, indels, tandem repeats, and structural variants) and gene expression differences among 14 inbred C57BL substrains. Genomic regions with dense polymorphisms between substrains are consistent with differential fixation or introgression rather than new mutations. They highlight significant associations between genomic variants and gene expression.

## Graphical Abstract

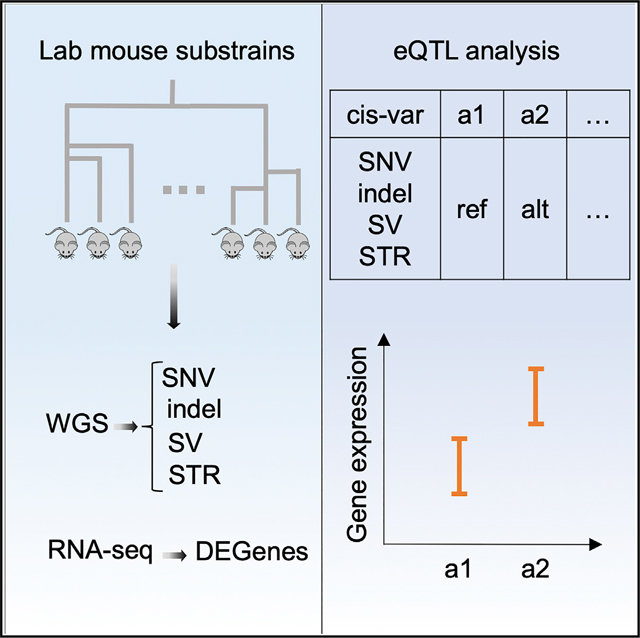

## INTRODUCTION

Since Clarence C. Little generated the C57BL/6 inbred strain a century ago, the C57BL/6J has become the most commonly used inbred mouse strain. Closely related C57BL/10 substrains,^1,2^ which were separated from C57BL/6 around 1937, are also commonly used in specific fields such as immunology^3^ and muscular dystrophy.^4^ The popularity of inbred C57BL strains has led to the establishment of many substrains (defined as >20 generations of separation from the parent colony). Among the C57BL/6 branches, the two predominant lineages are based on C57BL/6J (from The Jackson Laboratory [JAX]) and C57BL/6N (from the National Institutes of Health [NIH]^5,6^). Subsequently, several additional substrains have been derived from the JAX and the NIH branches.

Genetic differences between closely related laboratory strains have been assumed to be the result of accumulated spontaneous mutations.^7^ For those that are selectively neutral, genetic drift dictates that some new mutations will be lost, others will maintain an intermediate frequency, and others will become fixed, replacing the ancestral allele.^8^ Because of historical bottlenecks and small breeding populations, fixation of new mutations can be relatively rapid.

Numerous studies have reported phenotypic differences among various C57BL/6- and C57BL/10-derived substrains, which are likely attributable to genetic variation. For C57BL/6 substrains, these differences include learning behavior,^9^ prepulse inhibition,^10^ anxiety and depression,^11^ fear conditioning,^12–14^ glucose tolerance,^15^ alcohol-related behaviors,^16,17^ and responses to various other drugs.^18–21^ For C57BL/10 substrains, these differences include seizure traits^22^ and responses to drugs.^23^ Crosses between two phenotypically divergent strains can be used for quantitative trait mapping. Because crosses among closely related substrains segregate fewer variants than crosses of more divergent strains, identification of causal alleles is greatly simplified.^21^ Such crosses have been referred to as reduced complexity crosses (RCCs)^24^ and have been further simplified by the recent development of an inexpensive microarray explicitly designed for mapping studies that use RCCs.^25^

Whole-genome sequencing (WGS) technology provides a deep characterization of single nucleotide polymorphisms (SNPs), small insertions and deletions (indels), short tandem repeats (STRs), and structural variations (SVs). SNPs that differentiate a few of the C57BL/6 substrains have been previously reported.^21,26^ Although most SNPs are expected to have no functional consequences, a subset will, for example, SNPs in regulatory and coding regions, which can profoundly alter gene expression and function. STRs have never been systematically studied in C57BL substrains. STRs are highly variable elements that play a pivotal role in multiple genetic diseases, population genetics applications, and forensic casework. STRs exhibit rapid mutation rates of ~10^−5^ mutations per locus per generation,^27^ orders of magnitude higher than that of point mutations (~10^−8^),^28^ and are known to play a key role in more than 30 Mendelian disorders.^29^ Recent evidence has underscored the profound regulatory role of STRs, suggesting widespread involvement in complex traits.^30^ SVs include deletions, duplications, insertions, inversions, and translocations. SVs are individually less abundant than SNPs and STRs but collectively account for a similar proportion of overall sequence difference between genomes.^31^ In addition, SVs can have greater functional consequences because they can result in large changes to protein-coding exons or regulatory elements.^32^ Large SVs among C57BL/6 (but not C57BL10) substrains were identified using array comparative genomic hybridization^7^ and have also been identified in more diverse panels of inbred strains using WGS.^33^ Although some genetic variants that differ between closely related C57BL substrains have been previously reported,^7,34–36^ a comprehensive, genome-wide map of SNPs, indels, STRs, SVs, and gene expression differences among C57BL6 and C57BL10 substrains does not exist.

**Figure 1.**
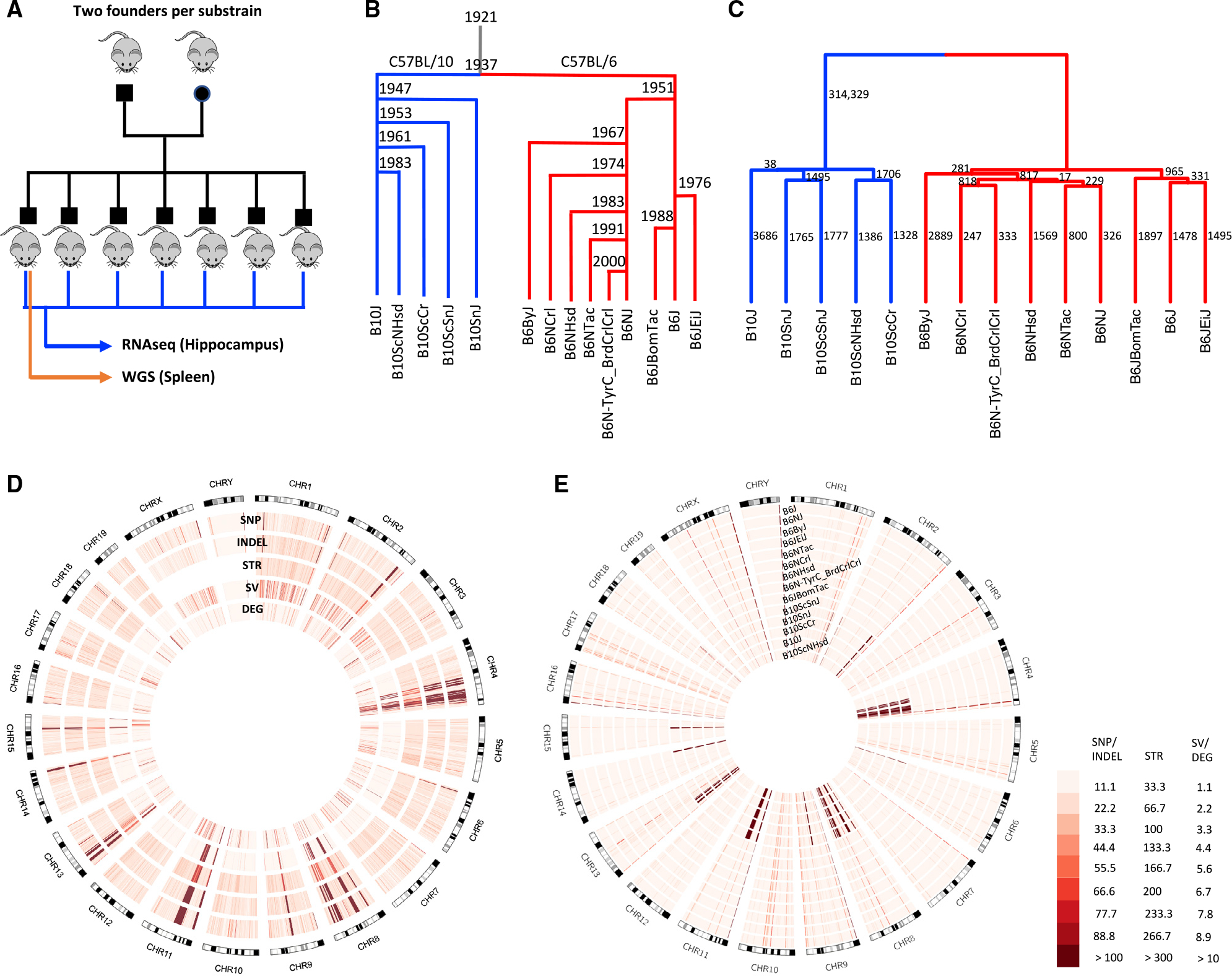
Study design, genetic distance analysis, and distribution of genomic variants across the genome (A) The design of our study. Mice from nine C57BL/6 and five C57BL/10 substrains were purchased from four vendors. For each substrain, between six and eleven male offspring from the first generation born in our colony were chosen for hippocampal RNA sequencing (RNA-seq). One male offspring per substrain was chosen for whole-genome sequencing (WGS) using DNA extracted from the spleen. (B) The historical relationship of C57BL/6 and C57BL/10 substrains is illustrated as a tree (Charles River Labs at https://www.criver.com/; Jackson Laboratory at https://www.jax.org/).^37^ (C) Dendrogram showing the similarity of the substrains based on genomic variants, including SNPs (LD-pruned, indels not included), STRs, and SVs. The numbers beside each branch indicate the number of SNPs separating the substrains in the subtree below the branch from all the other substrains. (D) Circos plot showing the SNPs, indels, STRs, SVs, and DE genes across the genome for 14 C57BL/6 and C57BL/10 substrains. Regions with a high density of polymorphisms (hot spots) on chromosomes 4, 8, 9, 11, and 13 are obvious (see also Figures S2 and S3). (E) Circos plot showing SNPs with non-reference genotypes for each substrain. This plot shows that most hot spots in (D) are due to regions where all C57BL/6 differ from all C57BL/10 substrains (see also Figures S2 and S3). A few regions where all substrains (including C57BL/6J) do not match the reference are also evident.

In an effort to create such a resource, we performed WGS in a single male individual from nine C57BL/6 and five C57BL/10 substrains (~30× per substrain) and called SNPs, indels, STRs, and SVs. In addition, to identify functional consequences of these polymorphisms, we performed RNA sequencing (RNA-seq) of the hippocampal transcriptome in 6−11 male mice from each substrain, which allowed us to identify genes that are differentially expressed (Figure 1A). This approach has two advantages: first it provides a large number of molecular phenotypes that may be caused by substrain-specific polymorphisms; and second, we assumed that the gene expression differences would often reflect the action of *cis*-regulatory variants, making it possible to narrow the number of potentially causal mutations without requiring the creation of intercrosses.

## RESULTS

Processing of WGS data from the 14 C57BL substrains (Table 1) allowed us to identify 352,631 SNPs, 109,096 indels, 150,344 polymorphic STRs, and 3,425 SVs in nine C57BL/6 and five C57BL/10 substrains; 5.6% of SNPs and 17.2% of indels are singletons (occur in only one substrain), and 89% of SNPs and 58% of indels separated the C57BL/6 and C57BL/10 branches. The fraction of variants in each category observed in different numbers of substrains is plotted in Figure S1.

RNA-seq analysis on 106 hippocampal samples identified 16,400 expressed genes and 2,826 differentially expressed genes (DE genes; 17.2%) in C57BL/6 and C57BL/10 substrains (false discovery rate [FDR] < 0.05).

RNA-seq data were also used to validate WGS SNPs and indels in protein-coding regions of the mouse genome (see STAR Methods, Figure S1, and Table S3). We observed a 97% validation rate for SNPs and 67% for indels.

Despite the lower sequencing depth on the X chromosome that was expected for male mice, we did not observe a difference in the density of private SNPs discovered on chromosome X compared with the autosomes (7.2 SNPs/Mb on X versus average 7.5 SNPs/Mb on the autosomes). However, the validation rate of coding SNPs on chromosome X was lower than on the autosomes (89% for chromosome X versus 97% on the autosomes).

We also detected 582,795 heterozygous SNP calls. For 92% of these heterozygous calls, all samples showed heterozygous genotypes, which suggests that most reflect segmental duplications (SegDups) rather than true heterozygous SNPs. Consistent with this idea, many apparently heterozygous SNPs were in known segmental duplication or tandem repeat regions (45%). For the remaining apparently heterozygous SNPs, we obtained a very low validation rate when using our RNA-seq data (12% validation rate for heterozygous SNPs in protein-coding regions that were not in known segmental duplication or tandem repeat regions; see also Table S3). Therefore, we did not include any heterozygous SNPs or indels in our analyses; however, we have included a VCF file with these variants in the supplemental information.

### Genetic evidence for origin of C57BL/6 and C57BL/10 substrain differences

Figure 1B shows the relationships among C57BL/6 and C57BL/10 substrains based on historical records (Charles River Labs at https://www.criver.com/; Jackson Laboratory at https://www.jax.org/).^37–39^ Figure 1C shows a dendrogram that was produced using SNPs, STRs, and biallelic SVs. The number of concordant SNPs separating the substrains in the subtree under each branch from all the other substrains is indicated beside each branch. A total of 342,002 SNPs (99.6% of non-monomorphic SNPs) agree with the dendrogram. Among 1,411 discordant SNPs, no particular substrain pattern is dominant. Comparison of Figures 1B and 1C shows that the records of the relationships among C57BL/6 and C57BL/10 substrains are consistent with our results.

### Distribution of genomic variants across the genome

The distribution of variants across the genome is shown in Figure 1D. Several dense clusters of variants common in all categories (SNPs, indels, STRs, and SVs) are evident (e.g., on chromosomes 4, 8, 11, and 13, for example). The non-uniformity of these polymorphisms is inconsistent with our expectation that polymorphisms among these substrains are due to new mutations and genetic drift. To further explore this observation, we examined the distribution of SNPs for each of the 14 different substrains. Figure 1E demonstrates that these clusters consist of a series of highly divergent haplotypes that differentiated the C57BL/6 and C57BL/10 lineages. In total, 312,981 SNPs (89% of SNP variants detected in this study and 99.6% of the C57BL/6 versus C57BL/10 SNPs) reside in C57BL/10-specific clusters that represent just 5% of the genome (28 segments on 11 chromosomes: 1, 2, 4, 6, 8, 9, 11, 13, 14, 15, 18) with a SNP density of ~1/425 bp. Across the remaining 95% of the genome, SNPs do not appear to be clustered and have a density of ~1/67,000 bp (more than 100-fold less dense). We found that many of the SNPs in these intervals are present in the Mouse Genome Informatics (MGI) database (http://www.informatics.jax.org/snp), which further suggests that they are not due to new mutations in the C57BL/10 lineage. We used the MGI database to identify strains that were similar to these 28 segments. No single strain matched all 28 segments. However, Figure S2 shows that, for 24 of the 28 segments, at least one strain in the database has greater than 90% concordance (we only considered strains for which a minimum of 300 SNPs were available in that segment). On the basis of these data, we hypothesize that one or more inbred or outbred mice were accidentally or deliberately introduced into either the C57BL/6 or C57BL/10 lineage. Another possibility is that their last common ancestor was not fully inbred and that these regions were differentially fixed after their separation. Most of the concordant strains that we identified in the MGI database have *domesticus* origin; however, two large segments on chromosomes 4 and 11 showed apparent *musculus* origin. We checked the subspecific origin of these 24 regions in C57BL/10J, C57BL/10ScNJ, and C57BL/10ScSnJ reported in Yang et al.^38^ The two large segments on chromosome 4 and 11 (with similarities to CZECHII/EiJ and MSM/Ms with *musculus* origins in the MGI database) showed *musculus* origins as well. Among the remaining 22 segments, all but three showed *domesticus* origins, and three segments (two on chromosome 4 and one on chromosome 11) showed some evidence of *musculus* origin.

Additionally, we identified 9,218 SNPs (2.6% of all SNPs) in which none of the C57BL/6 or C57BL/10 substrains matched the reference genome (mm10). One explanation is that some of these SNPs represent errors in mm10, perhaps related to the use of bacterial artificial chromosomes or other technical issues.^39^ Another explanation is that some of these SNPs represent true differences between the individuals used to generate mm10 and the individual C57BL/6J used for WGS in our study; we expect that there should be some unfixed polymorphisms within C57BL/6J that exist at intermediate frequencies, meaning that they will be observed in some individuals (e.g., the C57BL/6J individuals used to generate mm10) but not in other individuals (e.g., the C57BL/6J individual sequenced in our study).^40^ The distributions of other variants (indels, STRs, and SVs) mirrored SNPs and are plotted in Figure S3.

### Identification of candidate genomic variants causing differential gene expression

We found that 2,826 of 16,400 expressed genes (17.2%) were differentially expressed among the 14 substrains (FDR 0.05). We refer to these as DE genes. We assumed that many of the DE genes were due to local (*cis*) polymorphisms.

In order to identify genomic variants that might be causally related differential gene expression, we tested all identified variants (SNPs, indels, STRs, and SVs) in the *cis-*window (1 Mb upstream of gene start and 1 Mb downstream of gene end) for association with the corresponding DE gene. Specifically, we tested the association between the *cis-*variants and the median of DE gene expression by a linear regression test, using Limix.^41^ The resulting p values are reported in the supplemental information.

**Figure 2.**
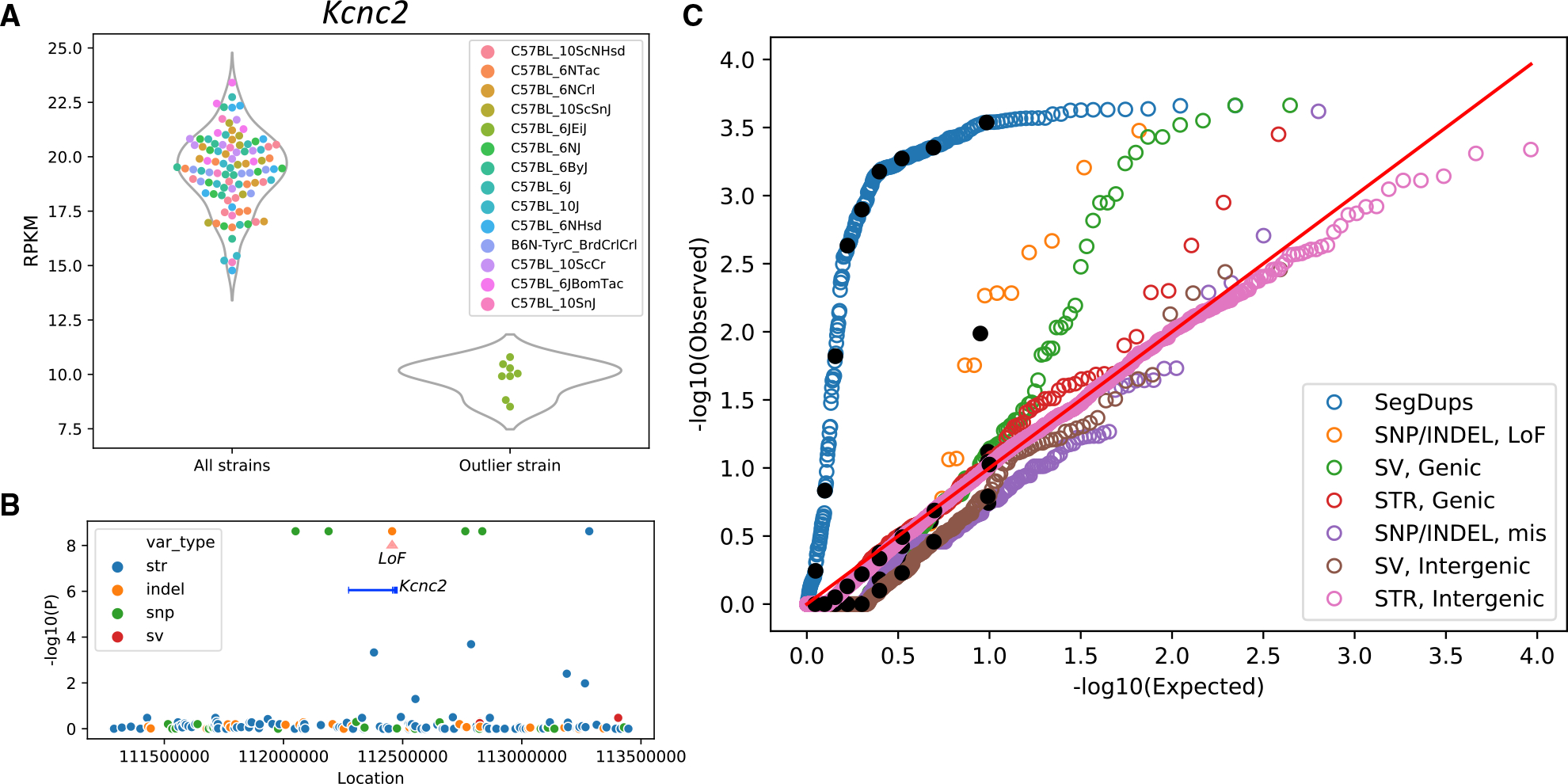
Association of gene expressions with genomic variants (A) Expression of *Kcnc2* is lower for C57BL/6JEiJ compared with the other substrains. (B) *cis*-variants of *Kcnc2* are tested for association with the median expression by the linear regression model. One indel is a frameshift loss-of-function variant and therefore has a strong prior to be the causal variant. In addition to that, four SNPs and one STR also have the same strain distribution pattern and therefore the same −log10 (p) value. For most of the DE genes, no variant belonged to a class that had a strong prior for causality. In those cases, any of the variants with the smallest p values (or a combination thereof, or more distant variants) might be causal (see also Figure S4). (C) The distribution of the p values of variants in different categories is compared against the uniform distribution in a QQ plot. A linear mixed model is used with the genomic relatedness matrix (GRM) as a random effect to control for population structure, and the parental strain (C57BL/6 or C57BL/10) is used as a fixed effect to identify associations within C57BL/6 and C57BL/10 substrains. The black dots show the deciles of the data in each category. The SegDup category includes associations between the copy number variation of the DE genes intersecting with SegDup regions (obtained by read depth across the segmental duplication regions of the reference genome) and the gene expression. Loss-of-function and missense mutations are two categories of SNP/indels. Genic SVs include those intersecting with gene features such as exons, TSSs, UTRs, promoters, enhancers, and introns, and genic STRs include those intersecting with exons, TSSs, 5’ UTRs, and promoters. Intergenic SVs and STRs are those not intersecting with any gene features and are paired with a gene with the closest TSS (see also Table S2).

As expected, all variants with the same strain distribution pattern have identical p values in the association tests. For example, for the gene *Kcnc2*, which had significantly reduced expression in C57BL/6JEiJ (Figure 2A), there was an equally strong correlation with four SNPs, one indel, and one STR in the *cis*-region (Figure 2B) and many more variants outside the *cis*-region (Figure S4). The indel was annotated as a frameshift loss-of-function variant by variant effect predictor (VEP);^42^ therefore, it had a strong prior to be the causal variant. Even in this small cohort of just 14 strains, we found a number of examples in which a variant within the *cis*-window had a strong prior and therefore appeared likely to explain a DE gene. We describe several such examples in the next section. However, for the majority of DE genes, there were no polymorphisms that had strong priors, meaning that any of the variants with the smallest p values in the *cis*-window, or a combination of them, or *trans-*acting variants elsewhere in the genome, could be causal.

### Differential expression of genes is associated with multiple categories of functional variants

Genomic variants that disrupt protein-coding exons or nearby *cis*-regulatory elements have the potential to cause differential gene expression. We investigated the causal role of variants in the *cis*-window by quantifying the strength of effects for multiple functional categories of variants. SNPs and indels were annotated using VEP,^42^ which identified 183 (58 SNPs and 125 indels) loss-of-function variants (frameshift, stopgain, or splice variant). We validated a random subset of these variants (13 SNPs and 29 indels) by Sanger sequencing (see STAR Methods and Table S4). All of the SNPs and indels showed 100% validation rates except for singleton indels, which showed a much lower validation rate of 42%. SVs were annotated by intersecting with the gene features including exons, transcription start site (TSS), untranslated regions (UTRs), promoters, enhancers, and introns. When a SV intersected with multiple types of functional elements, it was categorized according to the order mentioned above. The same gene annotations were applied to STRs. Intergenic SVs and STRs, which are defined as those that did not intersect with any gene features, were paired with the gene that had the nearest TSS. In addition, we assessed multiallelic copy number variation of genes by quantifying sequence coverage of all segmental duplications mapped to the mm10 reference genome that intersected with genes.

Genomic variants of the above-mentioned categories that intersected with DE genes were tested for association with gene expression by a linear mixed model (LMM) using Limix.^41^ We controlled for the complex relationships among inbred strains (a form of population structure) by using a genomic relatedness matrix (GRM), derived from SNP genotypes, as a random effect and parent strain (C57BL/6 or C57BL/10) as a fixed effect. Figure 2C shows the QQ plot for the p values obtained from the data versus the uniform distribution. The black dots show the deciles of the data in each category. SegDups that intersected genes were strongly correlated with the expression of those genes, as would be expected for gene copy number variation. Loss-of-function SNPs and indels also showed a significant inflation of correlated DE genes, followed by the genic SVs. The genic STRs showed a slight inflation, which was not as significant as other variant types. The missense SNP, intergenic SV, and intergenic STR p values followed the uniform distributions.

For each category, the p values obtained by the LMM are corrected by the Benjamini-Hochberg procedure to obtain a FDR. We identified 53 significant (FDR < 0.05) associations between DE genes and features, which are reported in Table S2. The majority of these associations (41 of 53 genes) reflected segmental duplications. In Table S2, we report the genotype pattern in the substrains for each variant as well; notably, there are several clusters of significant associations with the same genotype pattern. For example, one extensive region on chromosome 2 that clearly distinguishes C57BL/6NJ from all other substrains accounts for 18 of the 53 genes identified. Another cluster with a more complex pattern on chromosome 4 accounts for 11 of the 53 identified genes.

### Distinct mechanisms of differential gene expression caused by SVs

**Figure 3.**
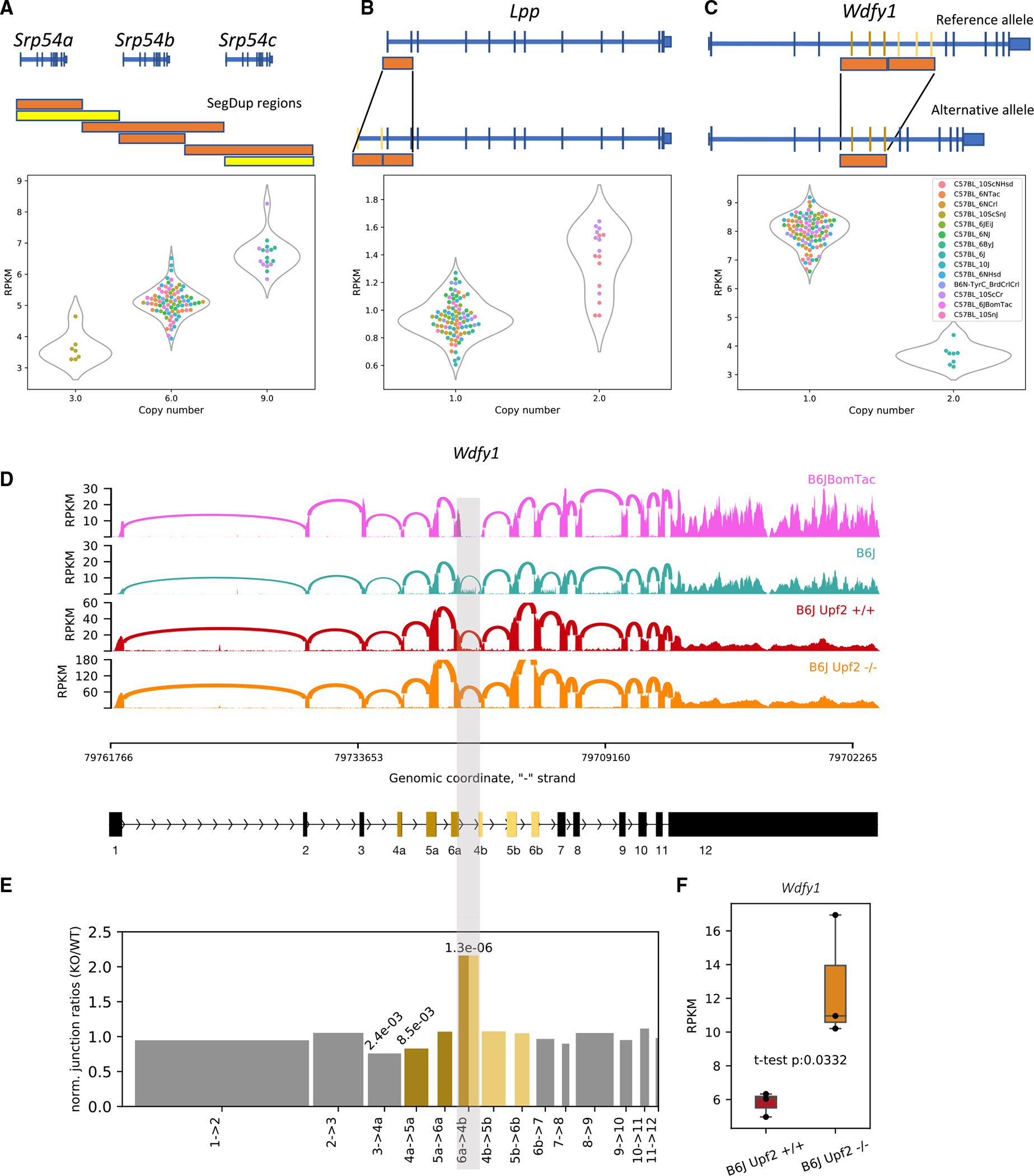
Structural variations affecting expression of *Srp54*, *Lpp*, and *Wdfy1* (A) Variation in copy number in a SegDup region is associated with expression of *Srp54*. The read coverage in these SegDup regions is used to infer the number of copies of the intersecting genes. The bars with similar lengths and colors indicate corresponding SegDup regions. Yellow bars indicate more than 98% sequence similarity; orange bars indicate more than 99% sequence similarity. (B) A duplication involving the first two exons of *Lpp* in two substrains of C57BL/10 is associated with an increase in *Lpp* expression. Duplication of the TSS and the promoter site is the most likely cause. (C) A segmental duplication region that intersects with three exons in *Wdfy1* and is present in C57BL/6J is associated with reduction of expression of *Wdfy1* in C57BL/6J. All the other substrains lack this duplication. (D) Sashimi plots^43^ for C57BL/6BomTac (a closely related substrain to C57BL/6J), C57BL/6J, *Upf2*^+/+^, and *Upf2^−^*^/−^ cell lines from C57BL/6J highlighting the junction between 6a and 4b exons across the segmental duplication region. Because C57BL/6BomTac lacks the duplication, it does not have any junctions between those exons, whereas the relative number of junctions in the *Upf2^−^*^/−^ cell line is significantly larger than the other wild-type C57BL/6J samples. (E) The bar plot shows the ratio of the normalized number of junctions between 6a and 4b exons (normalized by the total number of junctions in each sample)in *Upf2^−^*^/−^ over *Upf2*^+/+^ cell lines. It shows a significant increase in the relative number of junctions between the two segmental duplications in the *Upf2^−^*^/−^ cell line. The numbers on top of the bars show p values obtained by the chi-square test. (F) The expression level of *Wdfy1* in the *Upf2^−^*^/−^ cell line is significantly higher than in the *Upf2*^+/+^ cell line. This supports our hypothesis that the reduction of gene expression in C57BL/6J is due to the nonsense-mediated decay (NMD) mechanism.

SVs can affect gene function by (1) varying the dosage of a full-length gene, (2) deleting or inserting exons producing alternative isoforms of a gene, or (3) rearranging the *cis*-regulatory elements of genes. For example, there are three copies of the gene *Srp54* in the mouse reference genome, but we found significant variability in the number of copies across the substrains; the number of copies was strongly associated with expression of *Srp54* (Figure 3A). Thus, in this example, copy number variation in segmental duplication regions is the likely cause of differential gene expression. An example of a SV that likely impacts expression is the *Lpp* gene. The *Lpp* gene has a tandem duplication of the first two exons in two substrains (C57BL/10ScCr and C57BL/10ScNHsd) that creates two copies of the TSS, which probably accounts for its 2-fold increased expression (Figure 3B).

An intriguing example of altered expression caused by a SV is the *Wdfy1* gene. This gene has a tandem duplication of exons 4–6 in the C57BL/6J substrain, which is also present in the mm10 reference genome. We found that this duplication is associated with a paradoxical decrease in *Wdfy1* gene expression (Figure 3C), a result that could potentially be explained by nonsense-mediated RNA decay (NMD) of transcripts that contain duplications of exons 4–6. NMD is a highly conserved pathway that promotes the turnover of mRNAs harboring premature termination codons, including those generated by frameshifts.^44^ We found no evidence of unproductive transcripts *of Wdfy1* in Gencode VM23. However, we reasoned that if a major spliced isoform of *Wdfy1* contains tandemly duplicated exons, it could be detected in cells that are deficient in NMD. RNA-seq analysis of NMD-deficient (*Upf2^−^*^/−^) C57BL/6J embryonic stem (ES) cells showed that *Wdfy1* expression was increased 2-fold compared with control (sibling) C57BL/6J ES cells (Figure 3F). Analysis of splice junctions from RNA-seq confirmed the existence of an aberrant isoform in all C57BL/6J lines that includes splicing of exon 6 to the downstream (duplicated) exon 4 (Figure 3D), which we refer to as the “6→4b” junction. This splice junction was unique to C57BL/6J strains, and the ratio of 6a→4b to all splice junctions was increased by ~2-fold in the *Upf2^−^*^/−^ ES cells relative to control ES cells (Figure 3E). These results demonstrate that certain isoforms transcribed from *Wdfy1* in C57BL/6J mice are degraded by NMD and that the transcripts that are retained are alternative splice forms that exclude the 6a→4b junction.

## DISCUSSION

We performed a large-scale multi-omics analysis of 14 C57BL substrains. We identified 352,631 SNPs, 109,096 indels, 150,344 STRs, and 3,425 SVs; furthermore, of the 16,400 genes that were expressed in the hippocampus, 2,826 were significantly differentially expressed (FDR < 0.05). Unexpectedly, many of the polymorphisms that differentiated the C57BL/6 and C57BL/10 substrains were concentrated in a few haplotypes, comprising just 5% of the genome. These polymorphisms appear to be due to either introgression of an unrelated individual or incomplete inbreeding at the time that the C57BL/6 and C57BL/10 lineages diverged. Setting these introgressed regions aside, we tried to identify variants that were causally related to differential gene expression by focusing on the *cis*-regions around DE genes. This allowed us to identify 53 genes in which a variant with high prior probability to be causal was significantly associated with gene expression. While the majority of these 53 instances were caused by segmental duplications, several of which spanned many adjacent genes, a smaller proportion were due to SVs and indels (see Table S2). Inflation of test statistics for these categories of variants further underscores their likely causal roles; a relaxed FDR threshold would have identified more than 53 variant/DE gene associations.

An unexpectedly large subset of variants (89% of all SNPs) was concentrated in 28 highly diverged haplotypes that were present in all C57BL/10 strains and represented just 5% of the genome. These dense clusters of genetic variation (1 SNP/425 bp) perfectly differentiated C57BL/10 from C57BL/6, and likely reflect introgression from another strain. Intriguingly, the smaller haplotypes appeared to be of *domesticus* origin and were similar to haplotypes found in multiple non-C57BL inbred strains. The two largest haplotypes appeared to be of *musculus* origin and were also similar to multiple non-C57BL inbred strains. The exact sequence of events that led to this situation is impossible to deduce, but these patterns are clearly due to breeding events (intentional or accidental) rather than spontaneous mutations; this conclusion is based on several observations: (1) the density of the polymorphisms, (2) the abrupt boundaries of the regions/haplotypes, and (3) the fact that the SNPs in these introgressed regions are found in other inbred strains, which would not be the case if they were due to spontaneous mutations. A previous microarray study performed on 198 inbred mouse strains also identified SNP differences between C57BL/6J and three C57BL/10 substrains (C57BL/10J, C57BL/10ScNJ, and C57BL/10ScSnJ) for all the 28 introgressed segments that we identified;^38,45^ however, that study did not highlight the significance of that finding and did not have sufficiently dense coverage to define the boundaries of the introgressed regions. Although a majority of C57BL/10-specific genetic variants lie within these introgressed regions, they contained only a small fraction (~13%) of DE genes; however, given that the introgressed regions represent only 5% of the genome, this is still more than a 2-fold greater density of DE genes that would be expected if they were randomly distributed across the genome.

Outside of these apparently introgressed regions, we identified 37,745 SNPs that were distributed throughout the genome in a Poisson fashion with more than 100-fold lower density (~1 SNP/67,000 bp). These SNPs are apparently due to the accumulation of new mutations, and their identification was the original goal of our study. Dendrograms based on these SNPs recapitulated the historically recorded relationships among the substrains (Figure 1B). For the relatively large number of DE genes (>2,000) that were located outside of the introgressed regions, we considered the association between different categories of nearby (*cis*) variants and expression of DE genes. Variable copy number segmental duplicated regions were shown to be highly enriched for significant associations, as were genic SVs and loss-of-function SNP/indels (Figure 2C).

We presented several examples to highlight how different classes of variants underlie DE genes. For example, variable copy number segmental duplications led to both increased and decreased expression of *Srp54* (Figure 3A). In another example, duplication of TSSs led to increased expression of *Lpp* (Figure 3B). In the case of *Wdfy1*, duplication of several exons led to downregulation of expression (Figure 3C), which we showed was due to NMD-mediated mRNA decay (Figure 3D). *Wdfy1* was previously reported to be differentially expressed between C57BL/6J and C57BL/6NCrl and was identified as one of the candidate genes for reduced alcohol preference in C57BL/6NCrl.^46^ This gene is also within the QTL named *Emo4* (location: Chr1:68,032, 186–86,307,305 bp; http://www.informatics.jax.org/allele/MGI:3582656); mice that are homozygous for C57BL/6J allele are more active in the open field test. Whether *Wdfy1* is actually the cause of either association cannot be resolved by our study.

Despite the numerous examples in which likely causal variants were identified, a majority of the causal variants underlying DE genes remain unknown. Many are likely to be due to variants in regulatory regions that have not been distinguished from other nearby variants with the same strain distribution pattern (and thus identical p values). Although we focused on the possibility that DE genes were due to nearby variants (*cis*-eQTLs), the large fraction of differentially expressed genes (17.2% of all expressed genes) could indicate that many DE genes are due to *trans*-eQTLs. Producing crosses between pairs of strains will be necessary to address the relative importance of *cis-* versus *trans*-eQTLs in the observed DE genes; it is possible that such crosses could identify one or more major *trans*-regulatory hot spots.

Our results create a resource for future efforts to identify genes and causal polymorphisms that give rise to phenotypic differences among C57BL strains, using the increasingly popular RCC approach, in which two phenotypically divergent, nearly isogenic inbred substrains are crossed to produce an F_2_ population.^24^ Because of the low density of polymorphisms, identifying the causal allele is much more tractable. For example, the gene *Cyfp2* was identified as the cause of differential sensitivity to cocaine and methamphetamine in a cross between C57BL/6J and C57BL/6N.^21^ In the supplemental information, we have provided genomic variants (SNPs, indels, STRs, and SVs), DE genes in the hippocampus, and association tests between DE genes and nearby variants. In addition, we have provided the VEP-annotated SNP/indels, which distinguish loss-of-function, missense, and synonymous mutations. Our data also identify some regions that have a high density of polymorphisms that may complicate the RCC approach. For example, phenotypic differences between C57BL/6 and C57BL/10 strains might frequently map to the introgressed regions, which have a high density of polymorphisms that would significantly hinder gene identification and negate many of the advantages of RCCs. Furthermore, crosses between two C57BL/6 or between two C57BL/10 strains may map to large segmental duplication regions such as those on chromosomes 2 and 4 (see Figure 1E and Table S2), which would again hinder gene identification. Thus, one key observation from this study is that genetic differences among and between C57BL/6 and C57BL/10 strains are not uniformly distributed. Furthermore, our study used a single individual to represent each strain for WGS. Therefore, we did not explore the extent to which the polymorphic regions we identified may be segregating versus fixed within each inbred strain. If some of these polymorphic regions are not fixed, it would further complicate the analysis of RCCs.

Whereas the RCCs represent a forward genetic approach (starting with a phenotypic difference, searching for the genetic cause), another novel application of our dataset would be to select two strains that are divergent for a coding or expression difference and to use that cross to study gene function. This reverse genetic approach (starting with a genetic difference, searching for the phenotypic consequences) has not been attempted using closely related substrains but is conceptually similar to characterization of a knockout mouse. This approach is limited by the available polymorphisms. Although it would be necessary to account for the impact of linked polymorphisms, most of the polymorphisms would be unlinked and would not confound the interpretation of results.

In summary, we have created a dataset that elucidates the differences among C57BL strains and can be used for both forward genetic (RCC) and reverse genetic approaches. We identify previously unknown introgressed segments that differentiate the C57BL/6 and C57BL/10 lineages. Our results can also be used to explore mutational processes and highlight the tendency of inbred strains to change over time due to the accumulation of new mutations and genetic drift.

### Limitations of the study

This study has several limitations. First, only a single mouse per substrain was used for the WGS, meaning that some of the reported variants may not be fixed within a given substrain, and other unfixed mutations may not have been detected because they were heterozygous or not present in the single individual used for WGS. Because the RNA-seq used multiple individuals, some of the DE genes might have been due to these unfixed mutations, but the causal alleles for such DE genes would not have been detected by our analyses. Second, we performed RNA-seq for only a single tissue (hippocampus). This means that eQTLs that are specific to certain tissues, specific to less common cell types, or specific to certain developmental time points or environmental conditions or eQTLs with modest effect sizes were not detected. Third, we did not use long-read sequencing, which would have improved our ability to detect SVs. Another limitation of this study is that we used only male mice; therefore, sex-specific DE genes may have been missed. The female littermates were used for behavioral studies that we hope to publish in the future. Finally, because we did not perform any crosses, our association analyses implicated all of the variants that had the same strain distribution pattern as the DE gene in question. We assumed that most DE genes were due to *cis*-eQTLs, which limited out search space to nearby variants; however, it is possible that some of the DE genes were caused by distant polymorphisms (*trans*-eQTLs). Even after assuming that the causal variant was due to a *cis*-eQTL, there were typically multiple candidates, meaning that only variants with strong priors (e.g., SVs, loss of functions) could be confidently associated with a given DE gene. As a result, we could not confidently determine which variant was causal for the majority of DE genes. Addressing this problem would have required crosses, and even then, linkage disequilibrium would have made it very difficult to distinguish between putative *cis*-eQTL variants.

## STAR★METHODS

### RESOURCE AVAILABILITY

#### Lead contact

Further information and requests for resources should be directed to and will be fulfilled by the lead contact, Abraham A. Palmer (aap@ucsd.edu).

#### Materials availability

This study does not generate new unique reagents.

#### Data and code availability

- RNA-Seq and WGS short read raw data have been deposited at NCBI SRA and are publicly available as of the date of publication. Accession numbers are listed in the key resources table. All processed data have been deposited at Mendeley and are publicly available as of the date of publication. DOIs are listed in the key resources table.
- All original code has been deposited at Zenodo and is publicly available as of the date of publication. Accession numbers are listed in the key resources table.
- Any additional information required to reanalyze the data reported in this paper is available from the lead contact upon request.

### EXPERIMENTAL MODEL AND SUBJECT DETAILS

#### Mice

We obtained a panel of 14 C57BL substrains from four vendors. The panel included nine C57BL/6 substrains: C57BL/6J, C57BL/6NJ, C57BL/6ByJ, C57BL/6Ntac, C57BL/6JbomTac, B6N-TyrC/BrdCrCrl, C57BL/6NCrl, C57BL/6NHsd, C57BL/6JeiJ, and five C57BL/10 substrains: C57BL/10J, C57BL/10ScCr, C57BL/10ScSnJ, C57BL/10SnJ, C57BL/10ScNHsd (Table 1). All of the substrains were bred for one generation at the University of Chicago before tissue was collected for whole genome sequencing and RNA-sequencing; this avoided gene expression differences that were secondary to environmental differences among the four vendors. Mice were ordered in November 2014, arrived at University of Chicago in December 2014, and started breeding in January 2015. Tissues were extracted from the first generation at the age of 50–60 days old for RNA-sequencing. All procedures were approved by the University of Chicago IACUC. One hundred and ten male mice in total, with six to eleven mice per substrain, were chosen for RNA-sequencing from hippocampus, and one male mouse per substrain was chosen for whole genome sequencing from spleen (Figure 1A).

#### Whole-genome sequencing (WGS)

DNA from one male animal per substrain (n = 14) was extracted from spleens using a standard “salting-out” protocol. Sequencing libraries were prepared using a TruSeq DNA LT kit, as per the manufacturer’s instructions. Subsequently, sequencing data was generated by Novogene at an average depth of ~30x coverage on an Illumina HiSeq X Ten (paired-end 150bp) (Table 1).

#### RNA-sequencing

Total RNA was extracted from 110 hippocampal samples using Trizol reagent (Invitrogen, Carlsbad, CA). RNA was treated with Dnase (Invitrogen) and purified using Rneasy columns (Qiagen, Hilden, Germany). RNA-sequencing library prep and sequencing was performed by the University of California San Diego Sequencing Core using Illumina TruSeq prep and Illumina HiSeq 4,000 machine (single-end 50bp; Table 1).

#### Nonsense mediated decay assay

To determine whether SVs of the *Wdfy1* gene in C57BL/6J create novel mRNA isoforms that are degraded by the Nonsense-Mediated Decay (NMD) pathway, we performed RNA-Seq on mouse embryonic stem cells (mESCs) from a *Upf2^−^*^/−^ strain of C57BL/6J that has impaired NMD and control mouse mESCs from C57BL/6J. Samples with an RNA integrity index of >8 (as determined by a BioAnalyzer) were used for RNA-Seq analysis. The University of California San Diego Sequencing Core performed library preparation using ribosomal RNA depletion protocol followed by paired-end sequencing (100 cycles) using a HiSeq4000.

### METHOD DETAILS

#### RNA data processing

Reads were mapped to mouse reference transcriptome (mm10) using the splice-aware alignment software HiSat2,^47^ and counts were normalized using HTSeq.^48^ Only genes that had at least one Count Per Million (CPM), for at least two samples were included in our analysis. We further removed four outlier samples identified by PCA analysis. This left us with gene expression data for 16,400 genes across 106 samples in 14 substrains.

To identify Differentially Expressed Genes (DE genes) we performed analysis of variance using the *anova* function in R, and adjusted the p values by computing the false-discovery rate (FDR) using the *p.adjust* function in R, with the Benjamini-Hochberg procedure. We obtained 2,826 DE genes among C57BL/6 and C57BL/10 substrains combined, 1,210 DE genes within C57BL/6, and 104 DE genes within C57BL/10 substrains with FDR<0.05.

Reads from three replicates of *Upf2^−^*^/−^ samples and three controls from the NMD assay were mapped to the mouse reference genome (mm10) by HiSat2,^47^ and counts were normalized using HTSeq.^48^ We kept all genes with CPM>1 and normalized the counts with *edgeR*^49^ function in *R*, however, we only analyzed *Wdfy1* expression in an effort to detect differences in NMD between *Upf2^−^*^/−^ and control samples.

#### SNPs and indels

We used SpeedSeq^50^ to process the WGS paired-end reads. SpeedSeq uses BWA-mem (v.0.7.8) to map the reads to the mm10 reference genome, SAMBLAST^51^ to mark duplicates, Sambamba^52^ to sort the BAM files, and FreeBayes^53^ to jointly call SNPs and indels. indels are defined as insertions or deletions which are relatively short in length. The length range for the detected indels in our study is between one and 64 base pairs, which is approximately the lower bound for SV length scales. We restricted our analysis to variants that were fixed within individual substrains by including homozygous SNPs and indels only, resulting in a callset consisting of 352,631 SNPs and 109,096 indels. To assess validation rates of these variants, we utilized the RNA-Seq data to validate WGS variants in protein coding regions of the genome. Reads from samples in each substrain were combined and GATK best practices pipeline for RNA-Seq variant calling54 was used. Average genome-wide read coverage of RNA-Seq data for fourteen substrains ranged from 1.81× to 3.71× with a median of 3.08×. The reads were mapped to the reference genome (mm10) by STAR with 2-pass option.^55^ Subsequently, SplitHCigarReads command was used, followed by the base recalibration step. Afterwards, HaplotypeCaller was run on each substrain separately.

In each substrain, we validated a subset of WGS variants which were in the protein coding regions of the mouse genome (exons and UTRs obtained from Ensembl annotations), and had at least 3× coverage in our RNA-Seq data (24,260 SNPs, 5,637 indels).

Genotypes from the WGS data in the protein coding regions were compared with variants detected in RNA-Seq data, and validation rates for SNPs and indels as well as different categories of variants including C57BL/10-specific, singleton and monomorphic variants were shown in Figure S1 and Table S3. Overall, we observed 97% validation rate for the SNPs and 67% validation rate for indels. Among indel categories, C57BL/10-specific and monomorphic indels were among highest validation rates (72%) and singletons showed the lowest validation rate (52%). Information about the position of the validated SNPs and indels can be found in the Supplementary material.

In order to validate the 183 (58 SNPs and 125 indels) predicted loss-of-function variants by VEP,^39^ we performed Sanger sequencing for 13 randomly selected SNPs (7 C57BL/10-specific, 5 singleton, 1 monomorphic) and 29 randomly selected indels (9 C57BL/10-specific, 12 singleton, 8 monomorphic) (see Table S4). For each locus, we genotyped one sample from a randomly selected C57BL/10 substrain for C57BL/10-specific and monomorphic categories, or the substrain which had the singleton variant for the singleton category, and one C57BL/6J sample for control. The variants are provided in the Supplementary material.

When computing the identity-by-state (IBS) matrix for dendrograms, we LD-pruned the SNP panel with Plink^56^ (–indep-pairwise 50 5 0.5) yielding 16,739 SNPs. This pruned SNP set was augmented by STRs and all bi-allelic SVs, followed by computing the distance matrix with *dist* and plotting the dendrograms with *hclust* in *R* v3.6.1.

#### Short Tandem Repeat (STR)

We used HipSTR v0.6 with default parameters^57^ to call STRs from mapped reads using the mm10 reference STR set available from the HipSTR website (https://github.com/HipSTR-Tool/HipSTR-references). The reference STR set was generated using Tandem Repeats Finder^58^ allowing a maximum repeat unit length of 6bp. STRs for the substrains were jointly genotyped on a single node of a local server in batches of 500 STRs. Resulting VCF files from each batch were merged to create a genome-wide callset in VCF format. We filtered out calls with missing genotypes, as well as calls with reference alleles for all substrains, resulting in a total of 150,344 polymorphic STRs. The STR calls are available in the Supplementary material.

#### Structural Variations (SV)

SVs were detected using a combination of approaches. First, we called SVs with LUMPY^59^ and CNVnator,^60^ two complementary methods that rely on discordant and split read signals or coverage respectively. Second, because SV calling accuracy by the above methods is low in regions that are dense in segmental duplications, copy number variation within annotated segmental duplications was quantified directly from coverage, and these coverage values were used for the correlation of gene copy numbers with gene expression.

We filtered out SV calls that overlapped 50% or more with the gap regions of the mouse reference genome, as well as the calls with length smaller than 50 bp and larger than 1 Mbp. A more stringent >1,000 bp length filter was applied to CNVnator calls. We then filtered out non-homozygous calls and calls that were homozygous for the alternative allele in all substrains.

Concordant calls from LUMPY and CNVnator with 50% or greater reciprocal overlap and the same genotypes were merged and the breakpoints reported by LUMPY were used. Consensus calls that overlapped with annotated segmental duplications (SegDup) in the reference genome were excluded, and instead SegDup copy number was assessed directly from read depth signal using mosdepth v0.2.6^61^ with window size 100 bp. SegDup annotations from the mm10 genome with at least 98% similarity were intersected with gene annotations, and the median read coverage across SegDups which intersect with genes was normalized by the median coverage of the corresponding chromosome. These normalized coverage values were used to correlate gene copy numbers with gene expression. The final set of SVs included 3,425 deletions, duplications and inversions in nine C57BL/6 and five C57BL/10 substrains. The distribution of SVs in each category and substrain is summarized in Table S1. The VCF file of the SV calls, and the read coverage data for the SegDup regions are provided in the Supplementary material.

### QUANTIFICATION AND STATISTICAL ANALYSIS

The details regarding association analyses are presented in the results sections as well as in the figure legends, and the software information is presented in the key resources table. p-value of 0.05 after correction for multiple testing (Benjamini-Hochberg procedure) was used to identify significant associations. The t-test and Chi-squared tests in Figure 3 was performed by scipy module in python v3.

## Supplementary Material

1

2

3

## Figures and Tables

**Table 1.** Information about samples, WGS, and RNA-seq data used in this study

			Whole-genome sequencing		RNA sequencing	
				
Strain	Vendor	Strain ID	Reads	Coverage	Average reads	# samples

C57BL/6Ntac	Taconic	B6	336,470,599	33.65	13,459,345	8
C57BL/6NJ	JAX	#005304	333,094,625	33.31	15,767,477	9
C57BL/6NHsd	Harlan	#044	349,305,241	34.93	14,656,480	7
C57BL/6NCrl	Charles River	#027	301,990,053	30.20	19,408,390	8
C57BL/6JeiJ	JAX	#000924	442,604,628	44.26	16,334,723	8
C57BL/6JbomTac	Taconic	B6Jbom	309,053,911	30.91	17,340,684	7
C57BL/6J	JAX	#000664	326,208,826	32.62	18,555,108	8
C57BL/6ByJ	JAX	#001139	294,061,881	29.41	15,552,591	9
B6N-TyrC/BrdCrCrl	Charles River	#493	316,655,519	31.67	15,139,469	7
C57BL/10SnJ	JAX	#000666	284,191,454	28.42	14,012,862	6
C57BL/10ScSnJ	JAX	#000476	285,201,231	28.52	18,947,235	7
C57BL/10ScNHsd	Taconic	#046	326,586,425	32.66	12,453,407	11
C57BL/10ScCr	JAX	#003752	289,068,061	28.91	16,384,792	8
C57BL/10J	JAX	#000665	311,292,991	31.13	17,550,456	7
Average			321,841,818	32.18	16,013,858	7.86

**KEY RESOURCES TABLE:** 

REAGENT or RESOURCE	SOURCE	IDENTIFIER

Experimental models: Cell lines		

Mouse cell line: C57BL/6J *Upf2*-KO ES cells	Laboratory of Miles Wilkinson	N/A
Mouse cell line: C57BL/6J *Upf2*-WT ES cells	Laboratory of Miles Wilkinson	N/A

Experimental models: Organisms/strains		

Mouse: C57BL/6NTac	Taconic	Model No: B_6_
Mouse: C57BL/6NJ	The Jackson Laboratory	Stock No: 005304
Mouse: C57BL/6NHsd	Harlan	Order code: 044
Mouse: C57BL/6NCrl	Charles River	Strain code: 027
Mouse: C57BL/6JEiJ	The Jackson Laboratory	Stock No: 000924
Mouse: C57BL/6JBomTac	Taconic	Model No: B6JBom
Mouse: C57BL/6J	The Jackson Laboratory	Stock No: 000664
Mouse: C57BL/6ByJ	The Jackson Laboratory	Stock No: 001139
Mouse: B6N-TyrC/BrdCrCrl	Charles River	Strain code: 493
Mouse: C57BL/10SnJ	The Jackson Laboratory	Stock No: 000666
Mouse: C57BL/10ScSnJ	The Jackson Laboratory	Stock No: 000476
Mouse: C57BL/10ScNHsd	Taconic	Model No: 046
Mouse: C57BL/10ScCr	The Jackson Laboratory	Stock No: 003752
Mouse: C57BL/10J	The Jackson Laboratory	Stock No: 000665

Software and algorithms		

HiSat2 v.2.1.0	Kim et. al. 2019	http://daehwankimlab.github.io/hisat2/
HTSeq	Anders et. al. 2015	https://htseq.readthedocs.io/en/master/overview.html
SpeedSeq	Chiang et. al. 2015	https://github.com/hall-lab/speedseq
STAR v2.7.9a	Dobin et. al. 2013	https://github.com/alexdobin/STAR
GATK v4.2.2.0	Van der Auwera GA and O’Connor BD. 2020	https://gatk.broadinstitute.org/hc/en-us
Plink v1.9	Purcell et. al. 2007	https://www.cog-genomics.org/plink/
edgeR	Robinson et. al. 2010	https://bioconductor.org/packages/release/bioc/html/edgeR.html
HipSTR v0.6	Willems et. al. 2017	https://hipstr-tool.github.io/HipSTR/
LUMPY	Layer et. al. 2014	https://github.com/arq5x/lumpy-sv
CNVnator	Abyzov et. al. 2011	https://github.com/abyzovlab/CNVnator
Mosdepth	Pedersen and Quinlan 2018	https://github.com/brentp/mosdepth
Limix v3.0.4	Lippert et. al. 2014	https://horta-limix.readthedocs.io/en/api/installation.html
Variant Effect Predictor (VEP)	McLaren et. al. 2016	https://m.ensembl.org/info/docs/tools/vep/script/vep_download.html
sashimi_plot: used in Figure 3D	Katz et. al. 2010	https://miso.readthedocs.io/en/fastmiso/sashimi.html
R v4.1.1	R Core Team 2021	https://www.r-project.org
Custom codes required to generate figures and perform analyses in this paper	This paper	https://zenodo.org/record/5716285

Deposited data		

RNAseq and WGS short read data	This paper	SRA: https://www.ncbi.nlm.nih.gov/bioproject/PRJNA705216/
Data required to run our custom codes and reproduce the figures and results	This paper	Mendeley: https://doi.org/10.17632/39sw8xcrmv.2
Processed data generated in this paper	This paper	Mendeley: https://doi.org/10.17632/k6tkmm6m5h.3

## Highlights

- Detection of numerous polymorphisms among 14 inbred C57BL substrains
- A subset of regions with dense polymorphisms is not due to new mutations
- 2,826 genes showed significant differential expression among the C57BL substrains
- Association of differentially expressed genes with likely causal variants
