# Supplementary material for: SNPs, short tandem repeats, and structural variants are responsible for differential gene expression across C57BL/6 and C57BL/10 substrains
Source: Cell Genom. 2022 Mar 9;2(3):100102. doi: 10.1016/j.xgen.2022.100102 (PMC9205302; doi:10.1016/j.xgen.2022.100102)
Supplement: Document S1. Figures S1–S4 and Tables S1 and S2 [file mmc1.pdf]

Cell Genomics, Volume *2*

Supplemental information

SNPs, short tandem repeats, and structural variants are responsible for differential gene expression across C57BL/6 and C57BL/10 substrains

Milad Mortazavi, Yangsu Ren, Shubham Saini, Danny Antaki, Celine L. St. Pierre, April Williams, Abhishek Sohni, Miles F. Wilkinson, Melissa Gymrek, Jonathan Sebat, and Abraham A. Palmer

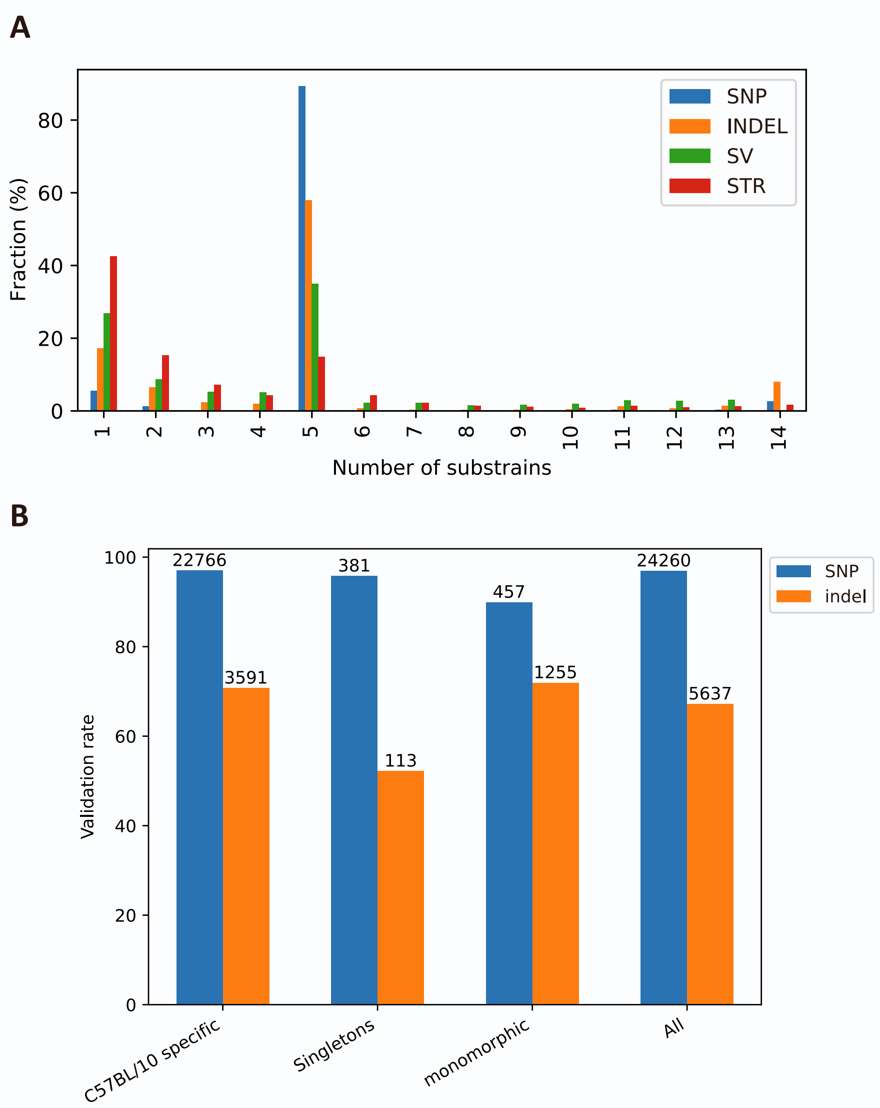

Figure S1. Variant frequency and validation rates, related to STAR Methods. A: Fraction of variants observed in substrains (five C57BL/10 and nine C57BL/6 substrains) in each variant category. The spike at 5 reflects polymorphisms that separated C57BL/10 (n=5) from C57BL/6 (n=9) substrains. The smaller spike at 14 represents instances where none of the substrains (including C57BL/6J, which is the basis for mm10) matched the mm10 reference genome. **B:** Validation rates of WGS variants in the protein coding regions using RNA-Seq data. WGS SNPs and INDELs which intersect with protein coding exon and UTR annotations (from Ensembl) and have at least 3X coverage in RNA-Seq dataset are considered for validation. Variants from RNA-Seq data were called by GATK best practices[S1] for each substrain separately (see also STAR Methods). Validation rate between different categories of variants are compared. The total number of variants in each category is indicated on top of each bar. Overall 97% of all SNPs and 67% of all INDELs were validated using RNA-Seq data.

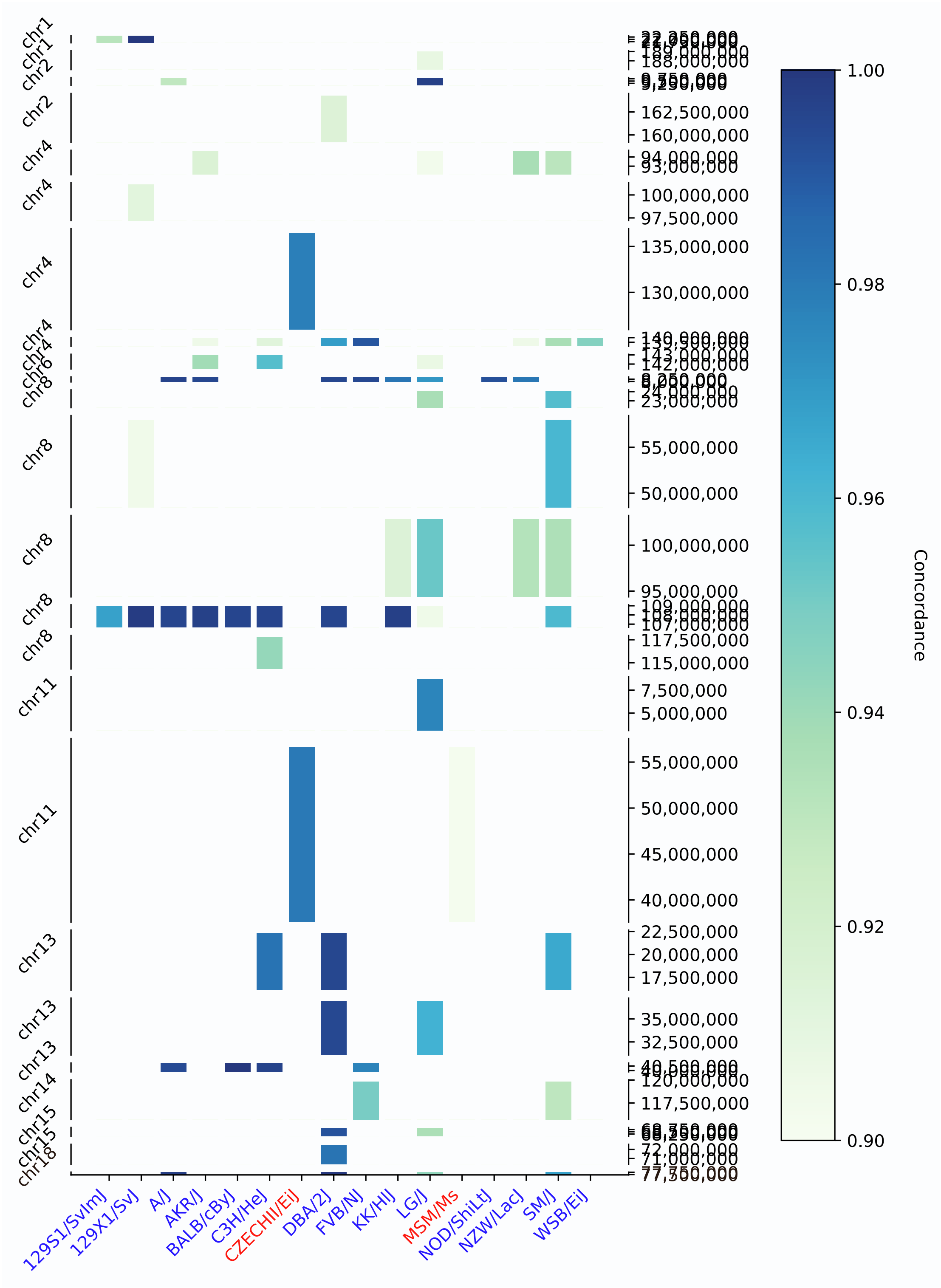

Figure S2. Concordance of C57BL/10-specific hotspot segments with other mouse strains, related to Figure 1. Concordance of 24 C57BL/10-specific haplotypes (SNP hotspots) with SNPs of other strains from *domesticus* and *musculus* origin. Y-axis shows

segments with C57BL/10-specific SNP hotspots. X-axis shows strains that have at least 300 common loci and at least 90% concordance with C57BL/10-specific SNPs in each segment. The SNP data for the strains is obtained from MGI[S2]. The segments are color coded with the concordance value. The strain labels on the x-axis are color coded with blue: *domesticus* origin, and red: *musculus* origin[S3].

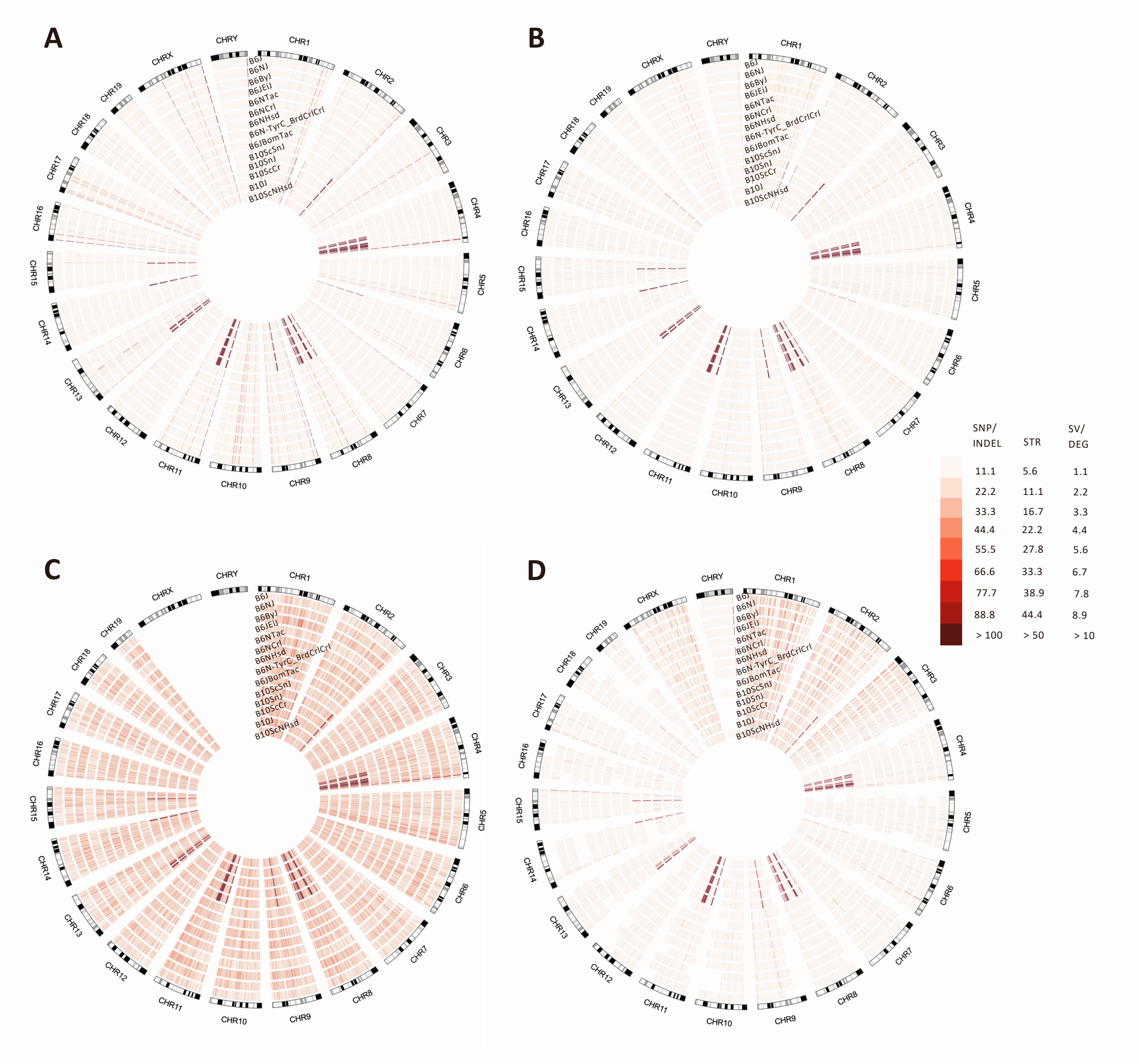

Figure S3. Distribution of genomic variations in 14 C57BL substrains, related to Figure 1. A: SNP distribution, **B**: INDEL distribution, **C**: STR distribution, and **D**: SV distribution for nine C57BL/6 and five C57BL/10 substrains show clusters of variants that are specific to C57BL/10 substrains on chromosomes two, four, eight, nine, eleven, thirteen, fourteen and fifteen.

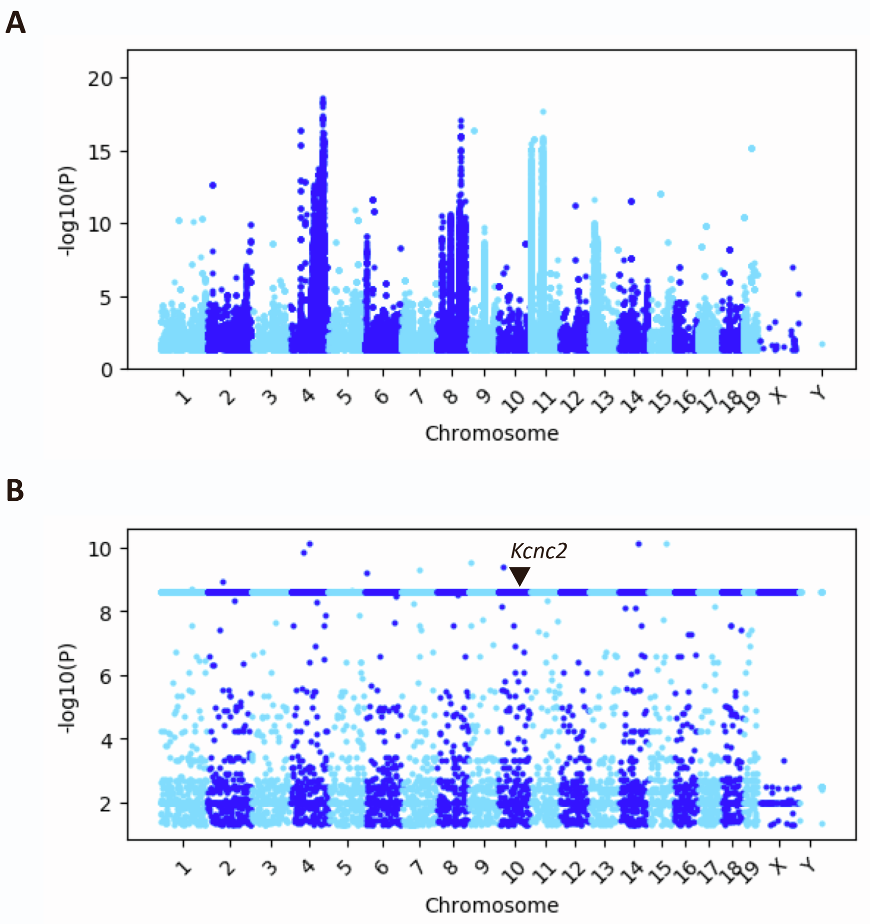

Figure S4. Association of all genomic variants and expression of DEGenes, related to Figure 2. Association tests of DEGene expressions of C57BL/6 and C57BL/10 substrains with all genomic variants (SNPs, INDELs, STRs and SVs) was performed by linear regression model with Limix[S4] **A**: Association of DEGene expressions with all variants (SNPs, INDELs, STRs and SVs) in the *cis*-region defined as 1Mb upstream and 1Mb downstream of the DEGene. The p-values are plotted at the genomic locations of the corresponding DEGenes. **B**: Association of *Kcnc2* expression with all genomic variants across the genome shows that variants with the same strain distribution pattern have identical p-values. The flat horizontal line at about -log10(p)=8.4 reflects features that have the same strain distribution pattern and therefore all yield identical p-values when tested for association with the gene expression data.

		CNVnator		Lumpy		
Strain	Substrain	DEL	DUP	DEL	DUP	INV
C57BL/6	C57BL/6J	27	461	11	97	2
	C57BL/6NJ	5	456	65	105	2
	C57BL/6ByJ	11	426	82	90	5
	C57BL/6JeiJ	14	469	42	91	4
	C57BL/6Ntac	27	465	64	77	4
	C57BL/6NCrl	9	420	66	92	2
	C57BL/6NHsd	127	488	73	86	3
	B6N-TyrC/BrdCrCrl	7	445	63	96	3
	C57BL/6JbomTac	13	447	50	78	3
C57BL/10	C57BL/10ScSnJ	95	430	1204	129	15
	C57BL/10SnJ	89	420	1195	125	13
	C57BL/10ScCr	269	481	1196	103	15
	C57BL/10J	74	437	1193	115	16
	C57BL/10ScNHsd	72	426	1192	123	14
	TOTAL	448	1308	1369	279	21

Table S1. Number of SVs found in C57BL substrains, related to Methods details.

Variant information	FDR	Gene
SV_DEL_chr4_66815109_66889841_exon	2.73E-02	*Tlr4*
SV_DUP_chr5_121767693_121843545_exon	2.73E-02	*Sh2b3*
SV_DUP_chr6_28420693_28493886_exon	3.05E-02	*Arf5*
SV_DUP_chr6_28420693_28493886_exon	3.21E-02	*Gcc1*
SV_DUP_chr16_24378934_24444153_exon	4.53E-02	*Lpp*
SV_DUP_chr19_36911370_37379559_exon	4.53E-02	*Btaf1*
SV_DUP_chr19_36911370_37379559_exon	2.73E-02	*Fgfbp3*
SV_DUP_chr19_36911370_37379559_exon	2.73E-02	*Ide*
INDEL_chr1_139237085_139237087_frameshift	2.02E-02	*Crb1*
INDEL_chr9_103499764_103499767_frameshift	4.25E-02	*Tmem108*
INDEL_chr10_112455818_112455821_frameshift	2.02E-02	*Kcnc2*
INDEL_chr16_10741835_10741842_frameshift	4.25E-02	*Clec16a*
SD_chr1_79715430_79722709	1.63E-03	*Wdfy1*
SD_chr2_174958961_175012684	1.09E-02	*Gm14444*
SD_chr2_175176236_175476161	3.35E-03	*Gm4631*
SD_chr2_175176236_175476161	1.63E-03	*Gm14391*
SD_chr2_175600000_175900699	2.60E-03	*Gm6710*
SD_chr2_175600000_175900699	1.95E-03	*Gm14288*
SD_chr2_175863477_175993394	4.45E-03	*Zfp966*
SD_chr2_176598872_176698447	2.38E-03	*Zfp973*
SD_chr2_176702042_176736143	1.67E-03	*Gm14305*
SD_chr2_176742479_176799992	1.63E-03	*Gm14295*
SD_chr2_176834443_177060914	1.63E-03	*Gm14296*
SD_chr2_176936335_177079562	2.50E-03	*Gm14401*
SD_chr2_177131439_177205055	1.63E-03	*Gm14410*
SD_chr2_177332425_177443112	1.63E-03	*Gm14418*
SD_chr2_177382170_177490852	1.63E-03	*Zfp970*
SD_chr2_177714087_177765961	1.91E-03	*Gm14322*
SD_chr2_177837513_177900857	1.63E-03	*Gm14325*

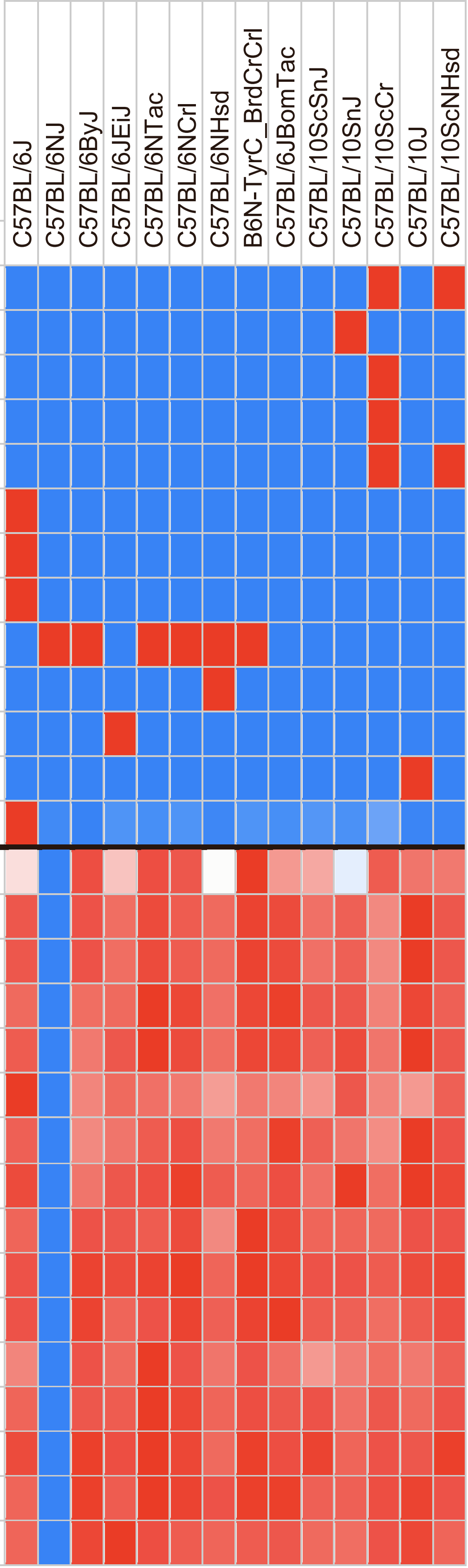

SD_chr2_177900735_177965213	1.63E-03	*Gm14326*
SD_chr2_177954479_178027444	1.63E-03	*Zfp971*
SD_chr4_41882892_42033952	1.63E-03	*Gm13302*
SD_chr4_41882892_42033952	1.63E-03	*Gm20878*
SD_chr4_41882892_42033952	1.63E-03	*Gm21093*
SD_chr4_41888084_42115916	1.63E-03	*Gm37530*
SD_chr4_41967677_42175681	3.35E-03	*Gm13306*
SD_chr4_42035272_42214541	1.63E-03	*Gm17167*
SD_chr4_42232133_42238731	1.63E-03	*Gm5859*
SD_chr4_42232133_42238731	1.63E-03	*Gm10600*
SD_chr4_42232133_42238731	1.63E-03	*Gm13301*
SD_chr4_42652191_42844101	1.63E-03	*Gm12394*
SD_chr4_42652191_42844101	1.34E-02	*Gm21955*
SD_chr12_55078703_55124935	1.63E-03	*Fam177a*
SD_chr12_55200000_55291223	1.63E-03	*Srp54c*
SD_chr14_6654813_6774339	1.63E-03	*Gm3636*
SD_chr14_6838781_6891591	4.45E-03	*Gm3667*
SD_chr14_7208412_7350351	3.87E-03	*Gm3739*
SD_chr17_6253548_6260484	3.14E-02	*Tmem181a*
SD_chr17_6325771_6473130	1.63E-03	*Tmem181b-ps*
SD_chr17_6497992_6645878	1.05E-02	*Dynlt1c*
SD_chr17_15014588_15045963	4.33E-03	*Ermard*
SD_chrX_169969770_170000000	2.95E-02	*G530011O06Rik*
SD_chrX_169969770_170000000	2.07E-02	*Gm15726*

Table S2. Significant associations and genotype patterns, related to Figure 2. Significant associations between DEGene expression and large effect variants with FDR<0.05 is presented. A linear mixed model is used with a Genomic Relatedness Matrix (GRM) to control for population structure as a random effect and parental strain (C57BL/6 versus C57BL/10) as a fixed effect to identify associations within C57BL/6 and C57BL/10 substrains. Variant information column indicates variant type (SD: segmental duplication), genomic coordinates and variant consequences (for SVs and INDELs). Colors represent genotype patterns for the variants. For bi-allelic variants (SVs and SNP/INDELs), red and blue colors represent two genotypes, while for multiallelic copy number variants the normalized read depth varies between 0 and 1 where 0: blue, 0.5: white, and 1: red represent three genotypes. The same genotype patterns are clustered together for chromosomes such as 2 and 4, which shows that nearby genes in these regions have been affected by the same copy number variation patterns. Bold horizontal lines segregate nearby variants with similar genotype patterns.

References:

[S1] Van der Auwera, G.A., and O'Connor, B.D. (2020). Genomics in the Cloud (O'Reilly Media, Inc.).

[S2] MGI Search Mouse SNPs. http://www.informatics.jax.org/snp.

[S3] Yang, H., Wang, J.R., Didion, J.P., Buus, R.J., Bell, T.A., Welsh, C.E., Bonhomme, F., Yu, A.H., Nachman, M.W., Pialek, J., et al. (2011). Subspecific origin and haplotype diversity in the laboratory mouse. Nature genetics *43*, 648-655.

[S4] Lippert, C., Casale, F.P., Rakitsch, B., and Stegle, O. (2014). LIMIX: genetic analysis of multiple traits. bioRxiv, 10.1101/003905.
